# Assessing subtypes of restricted and repetitive behaviour using the Adult Repetitive Behaviour Questionnaire-2 in autistic adults

**DOI:** 10.1186/s13229-018-0242-4

**Published:** 2018-11-26

**Authors:** Sarah L. Barrett, Mirko Uljarević, Catherine R. G. Jones, Susan R. Leekam

**Affiliations:** 1 Wales Autism Research Centre, School of Psychology, Cardiff University, Cardiff, UK; 2 Department of Psychiatry and Behavioral Sciences, Stanford University School of Medicine, Stanford, California USA

**Keywords:** Repetitive behaviours, Insistence on sameness, Repetitive sensory and motor behaviours, Adults, Principal components analysis, Questionnaire

## Abstract

**Background:**

The majority of previous research into restricted and repetitive behaviours (RRBs) has focussed on children, partly due to a lack of suitable measures for RRBs in adults. This study aimed to explore the psychometric properties of the Adult Repetitive Behaviour Questionnaire-2 (RBQ-2A) in a large sample of autistic adults using a self-report questionnaire method.

**Methods:**

The RBQ-2A and Autism-Spectrum Quotient (AQ) were administered online. Data from 275 autistic adults aged 18–66 (*M* = 36.56, SD = 12.24; 100 men and 171 women) were analysed using polychoric principal components analysis (PCA). Reliability and validity were assessed using Cronbach’s alpha and correlation analyses.

**Results:**

PCA resulted in two components of the RBQ-2A, interpretable as repetitive sensory and motor behaviours (RSMB) and insistence on sameness (IS). Both components showed acceptable internal consistency (*α* = .70 and .81 respectively) and were significantly moderately correlated with scores on the AQ (*r*_*s*_ = .25 and .42). Participants’ scores on IS were higher than their scores on RSMB. RSMB, but not IS, was negatively associated with age, particularly in older adults (≥ 50 years). There were no gender differences.

**Conclusions:**

The RBQ-2A is a reliable and valid self-report measure of RRBs in the present sample of autistic adults. As one of the few measures of RRBs aimed at adults, it is suitable for adults with the ability to read and complete a self-report questionnaire. Results build on previous work with children using the Repetitive Behaviour Questionnaire-2 (RBQ-2).

## Introduction

Restricted and repetitive behaviours (RRB) are an essential diagnostic criterion for autism spectrum disorder (ASD; [1]). RRBs encompass a wide variety of behaviours from motor stereotypes to maintaining order and sameness, and are seen across different situations and settings. This heterogeneous group of behaviours has been found to divide into two subtypes: Repetitive Sensory and Motor Behaviours (RSMB) and Insistence on Sameness (IS). This structure has been identified in factor-analytic studies of autistic populations [2–5] and typically developing (TD) and community populations [6–8]. However, some studies find between three and six subtypes [9–12], which may be a result of the use of different measures of RRBs, and the inclusion of children at different developmental stages in each study [13].

Previous research has focussed on RRBs in younger children, although they can be measured in adults [14, 15], including neurotypical (NT) adults [6, 16]. However, there are only two currently published measures of RRBs that have been developed with the aim of assessing RRBs in an adult population; the Adult Repetitive Behaviour Questionnaire-2 (RBQ-2A; [16]), and the Adult Routines Inventory (ARI; [6]). This paper aims to build on previous findings using the RBQ-2A by establishing the RBQ-2A’s reliability and validity in a larger sample of autistic adults, as well as exploring the presentation of RRBs in adulthood in relation to age and gender. It should be noted that the cognitive demands of completing a self-report questionnaire necessarily limits the representative nature of our sample to adults with competent levels of reading fluency and comprehension.

As previous research into RRBs has generally focussed on children, little is known about the expression and trends of RRBs across autistic adulthood, as well as how RRBs differ according to age and gender in autistic adults; therefore, we will consider some of the relevant findings in children as well as in adults. Although some previous factor analysis studies included adults, the age ranges of some of these studies are narrow and often limited to adults in their twenties (e.g. [17, 18]). Furthermore, there were no separate factor analyses of the adults alone. Only two studies have assessed the structure of RRBs in exclusively adult samples [6, 16], both of which found a two-dimensional structure corresponding to RSMB and IS. However, Barrett et al.’s [16] principal components analysis (PCA) was carried out on typical adults only, and Evans et al.’s [6] analysis was conducted on a large community sample that included adults with a range of neurodevelopmental and neuropsychiatric disorders as well as NT adults, with a low number of ASD individuals.

It is unclear the extent to which RRBs change with age in adulthood, although autistic traits in general are known to vary over time (e.g. [19]) and RRBs decrease in TD children after the age of 7 years (e.g. [8, 20]). There is evidence both for [15, 21] and against [22] the decrease of RRBs over time in autistic individuals. Esbensen et al. [14] reported a specific decrease in RSMBs with age compared to IS that was particularly pronounced in individuals with a learning disability (LD). Conversely, Georgiades et al. [2] found no difference in RSMBs between children and adults with ASD, but found that adults scored higher on IS. One study that exclusively assessed adults found no difference between older and younger adults with ASD [23], while another found that all RRBs decrease with age, particularly RSMBs [24]. Barrett et al. [16] did not find a significant association between age and RRBs in autistic or NT adults, although Evans et al. [6] found that all RRBs decreased with age in their cross-sectional sample of adults. Therefore, the precise relationship between age and RRBs is not clear in adulthood, although there is some evidence for IS behaviours being more common than RSMBs in adults.

In terms of gender, it has been shown that autistic women show significantly fewer RRBs than autistic men (e.g. [23, 25]). This has been supported in studies including children and adolescents [26]. There is some evidence that this varies according to RRB subtype and other factors such as concurrent mental health problems. For example, Evans et al. [6] found that while men scored higher on overall RRBs and RSMBs, there were no gender differences in terms of IS. Furthermore, in one study [27], girls and women scored higher in terms of IS as measured by the RBQ-2A compared to boys and men; this was potentially because the girls and women had higher self-reported anxiety, which is in turn positively associated with IS. However, our interpretation of this in relation to adults is limited by the inclusion of adolescents (14–18 years). In contrast, Barrett et al. [16] found no gender differences in RRBs, for either their NT or ASD group. The discrepancy in findings may be related to age, measurement and concurrent LDs. For example, all participants in Hattier et al.’s [23] study were diagnosed with LD, and they assessed RRBs using a caregiver-report measure, the Diagnostic Assessment for the Severely Handicapped [28], whereas Evans et al. [6] and Barrett et al. [16] used self-report measures and did not have exclusively LD samples. Moreover, the sample used by Uljarević et al. [27] focused on younger individuals with limited age range (15–25 years).

As the overview of the literature demonstrates, there is no clear consensus on the structure of RRBs in autistic adulthood and how it may differ according to age or gender. However, there are few dedicated measures to assess RRBs in adults to address these gaps. Although tools that have been employed to measure RRBs in autistic individuals, such as the Autism Diagnostic Interview-Revised (ADI-R [29]) and the Repetitive Behaviour Scale-Revised (RBS-R [30]) are reliable and valid, they are limited in other ways. For example, the ADI-R as a diagnostic instrument has been designed to be stable and reliable, but it samples a limited number of RRBs. Moreover, the ADI-R and RBS-R rely on caregiver-report which may be unreliable in the case of adults who have moved away from home, or unhelpful in the case of adults whose parents have passed away. Furthermore, observation measures, although reliable and valid in measuring RSMBs, are time-limited to the observational window and are limited in the measurement of IS behaviours [31].

The RBQ-2A was developed in response to the lack of suitable measures for assessing RRBs in adults and is based on the RBQ-2, a 20-item parent-report measure of the profile of RRBs in children. This measure was originally developed from the Diagnostic Interview for Social and Communication Disorders (DISCO [32, 33]) and the Repetitive Behaviours Interview (RBI [34]). The RBQ-2 demonstrates good reliability and validity, and a two-factor structure reflecting RSMB and IS. Strong psychometric properties and a stable factor structure have been reported in TD children at the ages of 15, 26, and 77 months (e.g. [8, 35]) and children and adolescents with ASD [36].

The adult version, the RBQ-2A, is similar to the child version with some questions reworded to be developmentally appropriate (see Barrett et al. [16] for details). The RBQ-2A was shown to be reliable and valid in a large sample of university students and a smaller sample comprising autistic and NT adults [16], as well as being reliable in adolescents and adults with ASD [27]. PCA of the university sample ([16] study 1) yielded a two-component solution; however, sensory items failed to load on either component. The two components were interpreted as IS and repetitive motor behaviours (RMB). This may be explained by the low endorsement of sensory items in the sample, reflecting that sensory symptoms are lower in NT compared to autistic individuals (e.g. [37, 38]). Conducting a PCA on a sample comprising only autistic adults will clarify the appropriate structure for the RBQ-2A.

The study presented here builds upon the findings of Barrett et al. [16] in two ways. Firstly, the study aimed to provide an additional assessment of the reliability and validity of the RBQ-2A by implementing PCA and assessing its correlational validity and internal consistency. It was expected that there would be two components corresponding to RSMB/RMB and IS as in previous research with the RBQ-2A. Secondly, we aimed to assess the profile of RRBs in autistic adults in terms of the different subtypes of RRBs and their differential association with gender and age, including differences between participants in early, middle and older adulthood.

## Method

### Participants

Participants were recruited through the Wales Autism Research Centre’s webpages, and several United Kingdom (UK) ASD charities. Only those who self-reported a clinical diagnosis of ASD and living in or originally from the UK (*N* = 352) were included in the study. Participants were aged between 18 and 66 years (*M* = 37.0, SD = 12.32), and comprised 132 men, 215 women and 5 individuals with a non-binary gender identity. The majority reported a clinical diagnosis of Asperger’s syndrome (*N* = 198; 56.3%). Most were white (92.6%) and lived in England (74.4%). Two hundred and seventy two (77.3%) participants had studied beyond the age of compulsory education in the UK (16 years). One hundred and forty-seven participants (41.8%) reported studying to degree level, and 66 participants (18.8%) had studied to postgraduate degree level. Two hundred and one participants (57.1%) were employed. Of the participants who reported the age of diagnosis (*N* = 341), 73.6% were diagnosed as an adult and 26.4% were diagnosed as a child. Since data collection was online, it was not possible to confirm diagnoses of participants; however, given there was no incentive to take part other than personal interest, it is unlikely that participants would dishonestly report a diagnosis of ASD. Nevertheless, participants were retained in the study only if their Autism-Spectrum Quotient (AQ [39]) score was at 26 or above [40].^1^ The lower cut-off of 26 was implemented in order to maximise the number of participants included in the study and to take into account autistic participants who may show lower levels of autistic traits. Removing participants scoring below 26 resulted in 309 individuals remaining in the sample. Online data collection procedures also prevented the opportunity to confirm IQ levels of participants; however, the level of education and employment and capacity for self-completion of the questionnaire were indirect indicators of a higher level of cognitive ability within the sample. Table 1 below summarises the demographic data for these participants.

**Table 1:** Summary of demographic information for participants from the UK meeting the Autism-Spectrum Quotient (AQ) cut-off of 26 (*N* = 309)

Age (years)	*M* = 37.44, SD = 12.44 Range: 18–66
Gender	116 (37.5%) male
	188 (60.8%) female
	5 (1.6%) non-binary
Reported diagnosis	179 (57.9%) Asperger’s syndrome
	78 (25.2%) Autism spectrum disorder (including ASD level one)
	32 (10.4%) High functioning autism
	20 (6.47%) Other^a^
Age at diagnosis	231 (74.8%) adult (18–66; *M* = 36.02, SD = 11.15)
	69 (22.3%) child (3–17; *M* = 11.62, SD = 4.16)
AQ score	*M* = 38.31, SD = 5.86, range: 26–50
Ethnicity	286 (92.6%) White
	22 (7.1%) mixed race, Asian, Black, Chinese or other
Location	230 (74.4%) England
	32 (10.4%) Wales
	43 (13.9%) Scotland, Northern Ireland or other
Education	Post-16: 239 (77.3%)
	Undergraduate degree 128 (41.4%)
Employed	171 (55.3%) employed
	137 (44.3%) unemployed

^a^Other includes atypical autism; autistic disorder; childhood autism; high-functioning autism/Asperger’s syndrome; pathological demand avoidance; Pervasive Developmental Disorder–Not Otherwise Specified

### Materials and procedure

The RBQ-2A comprises 20 items, scored on either a 3- or 4-point Likert scale. The fourth option is collapsed into option three while scoring [16]. A mean score across items is then calculated for each participant, with a maximum of 3. As with previous research, item 20 (*activities*) was not included in the PCA as it is qualitatively different from the other items [7, 36]. Although items 7 (*fascination*) and 12 (*collect*) are not included in studies with children due to failure to load in factor analysis studies, they were included here in order to test whether or not they load for autistic adults. The exact wordings of the 20 items comprising the RBQ-2A have previously been published [16].

The AQ is a 50-item self-report questionnaire that aims to assess traits and behaviours associated with ASD in the general population. Each participant receives a score out of 50, with higher scores indicating higher levels of autism traits. The AQ is not a diagnostic tool for ASD; however, it reliably distinguishes between autistic and NT individuals and shows good sensitivity and specificity [39, 40]. Demographic questions included age, gender, diagnostic information (specific diagnosis and age of diagnosis), educational level, employment, ethnicity, and country/region.

Participants completed the study online (via Google Documents), and received and completed the questionnaires in the fixed order of RBQ-2A; AQ; demographic questions. The study was approved by the Cardiff University School of Psychology Research Ethics Committee.

### Statistical analyses

As Likert scales produce ordinal rather than interval data, the recommended polychoric PCA (e.g. [41]) was carried out using the program FACTOR [42] to determine the structure of the RBQ-2A. Parallel analysis [43] was used to determine the number of components that should be retained. Direct oblimin rotation was employed to allow correlation between the two components. An item loading threshold of .4 was employed [44]. The remaining analyses were carried out using SPSS 20.0. Internal consistency was assessed by calculating Cronbach’s alpha (*α*) values. The correlational validity between AQ and RBQ-2A scores was assessed, along with the correlation between the RBQ-2A sub-scales. Finally, the effects of age and gender were explored.

## Results

### Descriptive statistics and data screening

Table 2 shows the endorsement, mean total scores and SDs for all 20 RBQ-2A items (*N* = 309). For every item, at least 18.2% of the sample endorsed one of the two higher response options (e.g. mild or marked). There were 34 (11.0%) participants with missing data across the relevant 19 items. Little’s Missing Completely at Random test was non-significant (*χ*^*2*^[369] = 400.39, *p* = .13) so it was appropriate to remove these participants from the analysis.

**Table 2:** Frequencies, percentages, means and standard deviations (SD) of participants’ responses to all twenty RBQ-2A items (*N* = 309)

	Never or rarely	Mild or occasional/one or more times daily	Marked or notable/15 or more times daily	Mean (SD)
1. Arrange	69 (22.3%)	182 (58.9%)	58 (18.8%)	1.96 (.64)
2. Fiddle^b^	38 (12.4%)	73 (23.8%)	196 (63.8%)	2.51 (.71)
3. Spin^a^	236 (76.6%)	56 (18.2%)	16 (5.2%)	1.29 (.56)
4. Rock^a^	125 (40.6%)	98 (31.8%)	85 (27.6%)	1.87 (.82)
5. Pace^a^	96 (31.2%)	121 (39.3%)	91 (29.5%)	1.98 (.78)
6. Hand/finger^b^	95 (30.9%)	83 (27.0%)	129 (42.0%)	2.11 (.85)
7. Fascination^b^	45 (14.7%)	117 (38.1%)	145 (47.2)	2.33 (.72)
8. Angles^d^	75 (24.6%)	144 (47.2%)	86 (28.2%)	2.04 (.73)
9. Smell^c^	132 (43.1%)	106 (34.6%)	68 (22.2%)	1.79 (.78)
10. Feel^d^	94 (30.8%)	105 (34.4%)	106 (34.8%)	2.04 (.81)
11. Carry^d^	124 (40.8%)	97 (31.9%)	83 (27.3%)	1.87 (.82)
12. Collect^b^	51 (16.6%)	93 (30.3%)	163 (53.1%)	2.36 (.75)
13. Home^b^	18 (5.9%)	65 (21.2%)	224 (73.0%)	2.67 (.58)
14. Change^c^	34 (11.1%)	88 (28.8%)	184 (60.1%)	2.49 (.69)
15. Routine^b^	20 (6.5%)	92 (30.0%)	195 (63.5%)	2.57 (.61)
16. Redoing^a^	16 (5.2%)	66 (21.4%)	226 (73.4%)	2.68 (.57)
17. TV/Music^b^	30 (9.8%)	83 (27.0%)	194 (63.2%)	2.53 (.67)
18. Clothes^d^	74 (24.3%)	106 (34.8%)	125 (41.0%)	2.17 (.79)
19. Food^b^	67 (21.8)	89 (29.0%)	151 (49.2%)	2.27 (.80)
20. Activities	11 (3.6%)	117 (37.9%)	181 (58.6%)	2.55 (.57)

Percentages given as valid percentages

^a^Missing = 1

^b^Missing = 2

^c^Missing = 3

^d^Missing ≥ 4

The final sample comprised 275 participants, aged from 18 to 66 years (*M* = 36.56, SD = 12.24; positively skewed [SW(275) = .96, *p* < .001] with no outliers), 100 of whom were men and 171 women. The total AQ score ranged from 26 to 50 (*M* = 38.51, SD = 5.88) and was positively skewed (SW[275] = .98, *p* < .001) with no outliers. There were 20 participants who had missing data on the AQ (ranging from 1 [2%] to 4 items [8%]). Little’s Missing Completely at Random test was non-significant (*χ*^*2*^[681] = 716.01, *p* = .17), so these participants were removed for analyses that included the total AQ score. The mean total RBQ-2A score ranged from 1.15 to 2.95 (*M* = 2.11, SD = .36), which was positively skewed (SW[275] = .98, *p* = .002) with no outliers. The internal consistency of all 20 RBQ-2A items was good (*α* = .84). The mean total RBQ-2A score significantly correlated with AQ (*r*_*s*_ = .43, *p* < .001), but not with age (*r*_*s*_ = −.01, *p* = .84).

### Principal components analysis

Initial screening indicated that the assumptions of sampling adequacy (KMO = .87), multicollinearity and factorability (*χ*^*2*^[171] = 1437.8, *p* < .001) were all met. The initial PCA solution resulted in four components with eigenvalues greater than 1, explaining 62.9% of the variance. Parallel analysis indicated that two components should be retained and the PCA was re-run with Direct Oblimin rotation. The correlation between components was .35, suggesting that oblique rotation was appropriate [45].

The final rotated solution explained 49.09% of the variance. Table 3 shows the pattern matrix of item loadings following Direct Oblimin rotation, along with the percentage of variance explained, Cronbach’s alpha value and mean inter-item correlation for each component. The analysis was re-run removing participants who did not score 32 or above on the AQ, in line with the original cut-off for this questionnaire [39]). The results of the analysis were broadly similar, with the exception that items 1 (*arrange*) and 11 (*carry*) loaded onto RSMB rather than IS. Given the lack of differences between the two solutions in terms of percentage of variance explained and reliability, the original analysis was retained in order to preserve sample size and to take into account autistic adults who may score lower on measures of autistic traits; this is particularly important given the large proportion of women and individuals diagnosed as adults who took part in the study.

**Table 3:** Pattern matrix for polychoric PCA after Direct Oblimin rotation (*N* = 275)

Rotated item loadings:	Component 1	Component 2
	Insistence on Sameness (IS)	Repetitive sensory and motor behaviours (RSMB)
1. Arrange	*.402*	.377
2. Fiddle	.038	*.783*
3. Spin	− .156	*.800*
4. Rock	−.052	*.815*
5. Pace	.036	*.609*
6. Hand/finger	.051	*.709*
7. Fascination	*.537*	.247
8. Angles	.368	.361
9. Smell	.298	.254
10. Feel	.258	*.490*
11. Carry	*.441*	.389
12. Collect	*.670*	.128
13. Home	*.885*	− .140
14. Change	*.844*	− .115
15. Routine	*.722*	.025
16. Redoing	*.801*	− .042
17. TV/music	*.496*	.341
18. Clothes	*.493*	.311
19. Food	*.523*	.052
Percentage of variance explained:	37.6%	11.5%
Cronbach’s alpha (*α*):	.81	.70
Mean inter-item correlation:	.29	.29

Items in italics met the item threshold (>.4) for that component

Table 4 shows the means and SDs, and the medians and IQRs for each component. The first component corresponds to IS, and the second component corresponds to RSMB. The internal consistency (Cronbach’s alpha) of IS was good (.81) and acceptable for RSMB (.70). The mean inter-item correlation for both components was .29.

**Table 4:** The ranges, means and SDs, and medians and IQRs for RSMB and IS (*N* = 275)

	Range	Mean (SD)	Median (IQR)
RSMB	1.0–3.0	1.97 (.48)	2.0 (.67)
IS	1.0–3.0	2.36 (.41)	2.45 (.55)

### Subscale analyses

Both components were positively skewed. There was one outlier who scored more than three SDs below the mean on IS; however, removing this participant from the analyses did not affect the pattern of findings. A Wilcoxon signed-rank test demonstrated a significant difference between IS and RSMB (*Z* = − 11.0, *p* < .001, *r* = − .66) with a large effect size.

Table 5 shows the correlations between the two components of the RBQ-2A, the AQ and age. Spearman’s correlation analyses indicated significant associations between RSMB and IS (*r*_*s*_ = .47, *p* < .001), and both components were significantly correlated with total AQ score. Only RSMB significantly correlated with age, and this association was negative (*r*_*s*_ = −.21, *p* < .001).

**Table 5:** Correlations between the subscales of the RBQA, the AQ and age (*N* = 275)

	RSMB	AQ (*N* = 255)	Age
RSMB	–	.30^a^	−.21^a^
IS	.47^a^	.44^a^	.06

^a^Significant at the .01 level

Table 6 shows the means, SDs, medians and IQRs for the sample when divided into three age groups (18–34; 35–49; 50–66) based on an approximate tertile split. Kruskall-Wallis tests indicated that there was a significant difference between age groups in terms of RSMB (*χ*^*2*^(2) = 12.34, *p* = .002) but not IS (*χ*^*2*^(2) = 1.67, *p* = .43). Follow-up Mann-Whitney tests indicated significant differences between 18 and 34 and 50–66-year-olds (*Z* = − 3.64, *p* < .001, *r* = − .27) and between 35 and 49 and 50–66-year-olds (*Z* = − 2.46, *p* = .014, *r* = .20), but not between 18 and 34 and 35–49-year-olds (*Z* = − .86, *p* = .39).

**Table 6:** Means, SDs, medians and IQRs for RSMB and IS by age group (*N* = 275)

	18–34 years (*N* = 129)		35–49 years (*N* = 98)		50–66 years (*N* = 48)	
	Mean (SD)	Median (IQR)	Mean (SD)	Median (IQR)	Mean (SD)	Median (IQR)
RSMB	2.05 (.47)	2.0 (.67)	1.98 (.51)	2.08 (.83)	1.76 (.40)	1.83 (.63)
IS	2.33 (.43)	2.45 (.55)	2.41 (.40)	2.5 (.64)	2.37 (.38)	2.45 (.45)

Finally, there were no significant gender differences in terms of the RBQ-2A subscales (Table 7). However, in terms of the AQ, women (*M* = 39.22, SD = 5.77) scored significantly higher than men (*M* = 37.48, SD = 6.12) with a small effect size (Z = − 2.23, *p* = .026, *r* = −.14). The two groups did not differ by age (men: *M* = 37.27 years, SD = 13.66; women: *M* = 36.11 years, SD = 11.23; *Z* = − .61, *p* = .54).

**Table 7:** Means, SDs, medians and IQRs for the RBQ-2A and its subscales by gender

	Men (*N* = 100)		Women (*N* = 171)		
	Mean (SD)	Median (IQR)	Mean (SD)	Median (IQR)	
RSMB	1.95 (.49)	2.0 (.83)	1.98 (.47)	2.0 (.67)	*Z* = −.47 *p* = .64
IS	2.35 (.41)	2.45 (.52)	2.37 (.41)	2.45 (.55)	*Z* = −.36 *p* = .72

## Discussion

This is the first PCA of self-reported RRB data from a sample of autistic adults aged 18 to 66 years. We examined the factor structure of the RBQ-2A and also the differential effects of age and gender on RRBs. A two-dimensional structure was identified using polychoric PCA, corresponding to IS and RSMB. This is in line with most RRB research, including findings from a community sample of adults that included autistic adults [6]. Both components showed good reliability as well as being significantly associated with AQ, supporting the construct validity of the RBQ-2A. Age was found to be significantly associated with RSMB, but not IS, with RSMBs decreasing with age. In contrast to previous research, there were no significant gender differences in RRBs. Given the lack of research into RRBs in autistic adulthood, the discussion will focus on the comparison between the present study and the previous research using the RBQ-2A [16].

Overall, the PCA structure supports previous findings using a university student sample [16], but with some minor differences. In terms of the IS component, the present solution is the same as Barrett et al.’s analysis with two exceptions. Two additional items loaded on to IS: items 7 (*fascination*) and 1 (*arrange*), which may reflect the samples used. Neither fascination nor arrange were highly endorsed in Barrett et al.’s university student sample, whereas there were relatively common in the present ASD sample. Although ASD individuals show more marked RRBs compared to the general population (e.g. [46, 47]), the difference may be particularly pronounced for certain RRBs such as fascination with specific objects and arranging objects.

The current RSMB component resembles the component RMB [16] with the exception of the exclusion of arrange and inclusion of item 10 (*feel*). The loading of a sensory item (*feel*) led to the decision to name the component RSMB rather than RMB. Again, endorsement of feel was much higher in the current sample compared to that in Barrett et al.’s participants. As well as differing in diagnosis, Barrett et al.’s participants were generally younger than in the present study (although spanned a similar age range from 18 to 50) and drawn only from university students, while the present sample was more evenly distributed in age and drawn from the general population.

Notably, two of the items that load differently across studies (*fascination* and *arrange*) were identified as sensory items in a study using an autistic child sample [36]. A relevant similarity between the two solutions is that sensory items 8 (*angles*) and 9 (*smell*) failed to load in either study. The unstable loading of four of the RBQ-2A’s sensory items across the two studies may indicate an inherent weakness in the sensory items. Alternatively, this instability may reflect genuine structural differences in RRB between autistic and NT individuals. The RBQ-2A may not capture a wide enough range of sensory responses to detect variation in a typical sample. For example, the RBQ-2A’s sensory items mainly focus on sensory-seeking rather than sensory-avoidant behaviours. There are also some modalities not included in the RBQ-2A that are included in other measures such as the Glasgow Sensory Profile [48], which includes auditory, vestibular and proprioceptive modalities. Indeed, studies using other measures find a relatively wide range of sensory behaviours in NT samples (e.g. [48, 49]). Further research directly comparing the performance of the RBQ-2A’s sensory items in a large sample of autistic and NT individuals would be useful, as well as comparing the RBQ-2A to more specific measures of sensory processing.

Apart from noted caveats that warrant further research, the results indicate that the RBQ-2A is a stable and reliable measure of RRBs in adults. The PCA findings presented here also generally reflect factor analytic findings by previous researchers using the child parent-report RBQ-2 [7, 8, 36], particularly in terms of IS. Again, there are some minor differences. First, *arrange* usually loads on to RSMB rather than IS. Second, in previous studies, *angles* has always loaded onto RSMB, whereas *smell* has loaded onto both RSMB [7, 8] and IS [36]. These differences may reflect the fluid nature of some RRBs, which can reflect either RSMB or IS depending on the intent and experience of the individual. This may also explain why two items in the present study (*arrange* and *carry*) load differently depending on whether the AQ cut-off of 26 or 32 is used. The desire to arrange items could be seen as an interest in patterns (RSMB) or a desire to impose order on the world (IS); similarly, an individual could be motivated to carry an object around because of a sensory property (RSMB), or because of a compulsion (IS). Overall, these minor differences between solutions do not undermine the conclusion that both the RBQ-2 and RBQ-2A can be conceptualised as a two-dimensional measure of RRB, comprising two subtypes of RSMB and IS. The two-dimensional solution supports the majority of findings using both interviews such as ADI-R as well as questionnaire measures (e.g. [3, 5, 50]).

Given the lack of data on the presentation of RRBs throughout autistic adulthood, the effect of age and gender on RBQ-2A scores was also examined. RSMB, but not IS, was significantly negatively correlated with age, and the sample scored significantly lower on the RSMB overall. When broken down into subgroups, older adults (50–66 years) scored significantly lower on RSMBs compared to the younger groups (18–34 years and 35–49 years), which did not differ from each other. Previous research has shown that while RRBs reduce across the lifetime, they remain more stable than social and communication traits [14, 15, 22]. Evans et al. [6] found significant negative associations between age, overall RRBs and both subtypes of RRBs in a community sample that included adults with ASD, as well as other neurodevelopmental and psychiatric conditions. However, there is also some evidence that RSMBs are particularly associated with younger individuals (e.g. [14, 51, 52]). The present results support and extend these findings, showing that RSMBs do not only occur less often in autistic adults compared to IS behaviours, but this difference is particularly pronounced in older adults.

There were no significant gender differences on the RBQ-2A, which supports previous findings using the RBQ-2A in both autistic and NT participants [16]. However, one study [27] found that female participants scored higher than male participants on the IS subscale of the RBQ-2A; although as mentioned, this particular sample included adolescents and the girls and women in the group showed high levels of anxiety. In contrast, research with other measures of RRBs tends to find that autistic men score higher than autistic women (e.g. [23, 53]). This discrepancy could be interpreted in one of two ways; firstly, it may be that the RBQ-2A is more sensitive to RRBs in women and therefore women score higher in line with men. Secondly, it could be that unlike other measures the RBQ2-A is not biased towards men. This could be explored by assessing RRBs using more than one measure in men and women.

### Strengths and limitations

The present study is the first to analyse the component structure of a sample that only comprises autistic adults, using polychoric PCA. Polychoric PCA is recommended for ordinal data, which is the form most questionnaire data takes, and yet the majority of factor analyses in this area are based on Pearson’s correlation matrix, which assumes continuous data. However, future research using the RBQ-2A should employ confirmatory factor analysis, which would provide a stringent test of the structure of the RBQ-2A. An important future direction is to explore the performance of the RBQ-2A as a measure of RRBs in other neurodevelopmental and neuropsychiatric disorders in order to establish sensitivity and specificity for ASD. Another important direction for future research is to assess the developmental trajectory of the RBQ-2 and RBQ-2A from childhood to adulthood.

An important limitation of this study is the fact that the present sample is not representative of the adult autistic population. Given the online design of the study, it was not possible to ascertain IQ or cognitive ability. However, it is important to highlight the fact that it would have been difficult for an individual to access and complete this self-report questionnaire if they had a significant LD or a specific difficulty with reading fluency or comprehension, and many reported high education and employment. This indirectly indicates that the majority had high ability, and further research is needed to establish a more representative sample. Furthermore, the majority of the current participants were diagnosed as adults, whereas individuals with ASD are usually diagnosed as children [54]. Similarly, the majority of participants were female, whereas ASD is diagnosed much more commonly in boys and men (e.g. [55, 56]); although the gender ratio in ASD may be a result of gender bias in diagnostic tools, misdiagnosis of girls and women, or masking of symptoms [57]. However, this also represents a strength of the research as it allowed us to explore gender differences in adequate group sizes. Finally, the majority of the sample identified as white (93%), and the findings may not be representative of other ethnicities. The characteristics of this sample therefore prevent us from generalising the present findings to the wider autistic adult population. However, this should be addressed in future research, by developing the RBQ-2A to make it more accessible for use with a more diverse and representative sample.

All of the measures used in this study were self-report. Although it has been suggested that autistic individuals may struggle with insights into their own feelings and behaviours [58, 59], previous research demonstrates that autistic individuals complete self-report questionnaires in a reliable and valid manner [60, 61]. While comparisons between parent and self-completed questionnaires (RBQ-2 and a modified version of the RBQ-2A) have been conducted with autistic adolescents [62], future studies should collect RBQ-2A data from autistic adults and their relatives in order to check their correlational validity; although as noted earlier, parent-report may be less useful for reporting on adults compared to children.

Finally, despite the advantage of the online nature of the study allowing us to reach a large number of participants, there are some inherent limitations to online research. Notably, it was not possible to independently confirm diagnoses of ASD; participants were asked whether or not they had a clinical diagnosis of ASD, but they did not provide further details or evidence. Care was taken to only include participants who reported a clinical diagnosis of ASD and scored at 26 or above on the AQ, which has been shown to be a cut-off with good sensitivity and specificity for ASD [40]. Although the psychometric properties of the RBQ-2A have been assessed in a small number of autistic adults with confirmed diagnoses [16], these should be assessed in a larger sample.

## Conclusion

The study presented here was the first to explore the factor structure of RRBs in a sample solely comprising autistic adults. Results suggest RBQ-2A to be a two-dimensional measure comprising RSMB and IS. This study also found that RSMBs, but not IS, decrease with age in autistic adults. In contrast to previous research, we did not find evidence of gender differences in terms of RRBs. One issue that remains for future research is whether or not to use the structure determined here in autistic adults or the structure identified by Barrett et al. [16] in university students. Given the use of the target ASD population, polychoric PCA, and a larger sample size in this study, we would recommend using the structure identified here. However, it would be important to carry out confirmatory factor analysis in an independent sample to test the best solution, especially given the instability of the sensory and carry items. Furthermore, future research should address the demographic limitations of the sample here as a priority before generalising the findings to the wider ASD population. Finally, an important step for future research is to consider the trajectory of RRBs from childhood to adulthood, and how the content of RSMB and IS may change over time. Our results show that RRBs reduce over adulthood, but the subtypes remain similar to those seen in children. Overall, the results reported here highlight preliminary evidence of the usefulness of the RBQ-2A as a reliable and valid measure of RRBs in self-reporting autistic adults.

## Abbreviations

ADI-R: Autism Diagnostic Interview-Revised
AQ: Autism-Spectrum Quotient
ASD: Autism spectrum disorder
DISCO: Diagnostic Interview for Social and Communication Disorders
IQR: Inter-quartile range
IS: Insistence on Sameness
LD: Learning difficulty
NT: Neurotypical
PCA: Principal component analysis
RBQ-2: Repetitive Behaviour Questionnaire-2
RBQ-2A: Adult Repetitive Behaviour Questionnaire-2
RBS-R: Repetitive Behavior Scale-Revised
RMB: Repetitive motor behaviour
RRB: Restricted and repetitive behaviour
RSMB: Repetitive sensory and motor behaviour
SD: Standard deviation
TD: Typically developing
UK: United Kingdom

## Footnotes

1 One participant had a score of 24 but missing data on two items, so may have reached the cut-off of 26. However, as it was not possible to know what their scores would have been, they were not included in the analysis.
